# A closer look into close packing: pentacoordinated silicon in a high-pressure polymorph of danburite

**DOI:** 10.1107/S2052252517010612

**Published:** 2017-08-10

**Authors:** Anna Pakhomova, Elena Bykova, Maxim Bykov, Konstantin Glazyrin, Biliana Gasharova, Hanns-Peter Liermann, Mohamed Mezouar, Liudmila Gorelova, Sergey Krivovichev, Leonid Dubrovinsky

**Affiliations:** a Deutsches Elektronen-Synchrotron (DESY), Hamburg 22607, Germany; b Bayerisches Geoinstitut, University of Bayreuth, Bayreuth 95440, Germany; c Institut für Beschleunigerphysik und Technologie (IBPT), Karlsruhe Institute of Technology, Karlsruhe 76021, Germany; d European Synchrotron Radiation Facility, Grenoble Cedex 38043, France; e Institute of Earth Sciences, Saint Petersburg State University, Saint Petersburg 199155, Russian Federation

**Keywords:** phase transitions, polymorphism, five-coordinate silicon, danburite, silicates

## Abstract

Due to their high technological and geological relevance, silicates are one of the most studied classes of inorganic compounds. Under ambient conditions, the silicon in silicates is almost exclusively coordinated by four oxygen atoms, while high-pressure treatment normally results in an increase in the coordination from four- to sixfold. Reported here is a high-pressure single-crystal X-ray diffraction study of danburite, CaB_2_Si_2_O_8_, the first compound showing a step-wise transition of Si coordination from tetrahedral to octahedral through a trigonal bipyramid. Along the compression, the Si_2_O_7_ groups of danburite first transform into chains of vertice-sharing SiO_5_ trigonal bipyramids (danburite-II) and later into chains of edge-sharing SiO_6_ octahedra (danburite-III). It is suggested that the unusual formation of an SiO_5_ configuration is a consequence of filling up the pentacoordinated voids in the distorted hexagonal close packing of danburite-II.

## Abstract 2

High-pressure single-crystal X-ray diffraction study of danburite, CaB_2_Si_2_O_8_, has revealed that it is the first compound showing a step-wise transition of Si coordination from tetrahedral to octahedral through trigonal bipyramidal. Along the compression, the Si_2_O_7_ groups of danburite first transform into chains of vertice-sharing SiO_5_ trigonal bipyramids (danburite-II) and later into chains of edge-sharing SiO_6_ octahedra (danburite-III).

## 1. Introduction   

Silicates are widely used in different chemical processes ranging from catalysis to cement production. As the most common minerals in the Earth’s crust and mantle, silicates have attracted significant attention since the beginning of structural crystallography. Due to the high geological relevance of silicates, numerous X-ray diffraction studies have been performed in order to understand their crystal structures as a function of chemical composition, temperature and pressure.

Modern crystal chemistry of silicates is based on the idea of nearly exclusive fourfold coordination of silicon under ambient conditions. Polymerization of SiO_4_ tetrahedra results in the formation of a very high number of different silicate anions that constitute the basis of silicate crystal structures. While Pauling (1929 ▸) gave rules to describe the principles of structural organization of ionic compounds under ambient conditions, several ‘rules of thumb’ were outlined by Prewitt & Downs (1998 ▸) to address the high-pressure evolution of silicates. ‘Increasing pressure increases coordination number’ is one of them, reflecting the observed tendency of silicon to change its coordination from tetrahedral to octahedral upon compression (Finger & Hazen, 2000 ▸). Indeed, the crystal chemistry of rock-forming silicates with four- and six-coordinated silicon is well established, whereas only a very few examples of structures with five-coordinated Si are known. These examples are limited to cases where SiO_5_ groups co-exist with SiO_4_ tetrahedra and/or SiO_6_ octahedra. Distorted SiO_5_ square pyramids along with SiO_6_ octahedra were found in the crystal structure of the titanite-like silicate CaSi_2_O_5_ (Angel *et al.*, 1996 ▸). Recently, high-pressure polymorphs of inosilicate pyroxenes have been considered as possible carriers of SiO_5_ structural units. Thus, Finkelstein *et al.* (2015 ▸) reported two new phases displaying silicon in a nearly fivefold coordination. In that work, Si was not described as fully pentacoordinated because of the high level of distortion of the SiO_5_ polyhedra: the four conventional ‘tetrahedral’ bond lengths varied in the range 1.58–1.69 Å, while the fifth exceeded 1.92 Å. Pentacoordinated silicon with highly distorted coordination polyhedra was also mentioned in the structure of dehydrated brewsterite (Alberti *et al.*, 1999 ▸). Here, we report a high-pressure single-crystal X-ray diffraction study of danburite, CaB_2_Si_2_O_8_, the first compound showing a step-wise transition in Si coordination from tetrahedral to octahedral through a trigonal bipyramid.

## 2. Materials and methods   

The sample of danburite originated from the Dalnegorsk boron deposit (Primorsky Krai, Russian Federation) (Karas *et al.*, 2008 ▸). BX90 diamond anvil cells (DACs) were used for pressure generation (Kantor *et al.*, 2012 ▸). The sample chamber was obtained by drilling a preindented rhenium gasket. Danburite crystals were placed inside the sample chambers, along with ruby spheres to enable pressure estimation (Mao *et al.*, 1986 ▸). The DACs were loaded with a neon pressure-transmitting medium (Kurnosov *et al.*, 2008 ▸). Monochromatic single-crystal X-ray diffraction experiments were performed on beamline P02.2 at PETRA III (DESY; experiment 1) and on beamline ID27 at the European Synchrotron Radiation Facility (ESRF; experiment 2). At each pressure both a wide scan and a stepped ω scan were collected for each crystal. Wide scans consisted of 40 s exposure during rotations of ± 20° of the DAC. Step scans were performed with individual exposures taken over 0.5° intervals to constrain the ω angle of maximum intensity of each peak. Collected diffraction images were analysed using the program *CrysAlisPRO* (Agilent, 2012 ▸). The *SHELXL* program package was used for all structural determinations (Sheldrick, 2015 ▸).

The structure refinements performed at 25.4 (1) GPa for danburite-II and danburite-III in experiment 1 are of the best quality and therefore they are used for the following crystal chemical discussion. Equation-of-state fits were performed with the *EoSFit7c* program using the Murnaghan equation of state (Angel *et al.*, 2014 ▸). For full details of the structure refinement, see the supporting information.

High-pressure Raman and IR spectroscopy measurements were performed on beamline IR2 of the ANKA synchrotron facility at Karlsruhe Institute of Technology. The same strategy of DAC preparation was applied, with the only difference being the usage of type IIa diamonds to ensure high transparency for the IR experiment. The IR spectra were collected with a Vertex80v FT–IR spectrometer coupled to an IRscopeII microscope in transmitted-light mode using Schwarzschild objectives (15×, 0.5 N.A.), an aperture of 30 µm and a liquid-N_2_-cooled MCT detector. The Raman spectra were collected in the range 100–1220 rel. cm^−1^ in back-scattering geometry using a WITec alpha300 confocal Raman system. A 532 nm frequency-doubled Nd-YAG laser was used, coupled *via* a single-mode fibre to a microscope with a 50× long working distance objective (N.A. = 0.5). The scattered light was sent to the spectrometer *via* a multi-mode fibre and recorded on a CCD detector. The collected spectra are given in the supporting information.

It is important to note that the high-pressure transformation route of danburite is sensitive to the presence of deviatoric stresses within the pressure chamber. In the course of experiment 1, danburite-II and danburite-III were found simultaneously at 25.4 (1) GPa, while in experiment 2 we clearly observed a step-wise character of these transformations (Fig. S1). In addition, the transition of danburite-II into a *P*2_1_/*c* phase with the β angle deviating slightly from 90° (danburite-IV) was detected above ∼32 GPa in the course of experiment 1. We assume that the observed differences in transformation routes originated from the large thickness of the crystal (>20 µm) that bridged between the diamonds at the transition pressures in experiment 1. This feature makes it difficult to define the assignment of the vibrational bands to either five- or eight-coordinated silicon above the danburite → danburite-II transition pressure. Thus, the characteristic Raman band for *T*—O—*T* stretching disappears at 25 GPa, and instead a new band appears at 640 cm^−1^ at 25.5 GPa and moves to 655 cm^−1^ at 34 GPa, which most probably reflects the formation of SiO_6_ octahedra (Goryainov, 2016 ▸) and/or SiO_5_ trigonal bipyramids. The abrupt change in the IR spectrum above ∼23 GPa also reflects the increase in Si coordination, which is manifested by the appearance of many new bands in the region 600–900 cm^−1^.

## 3. Results   

### 3.1. Crystal structure of danburite under ambient conditions   

**Figure 1:**
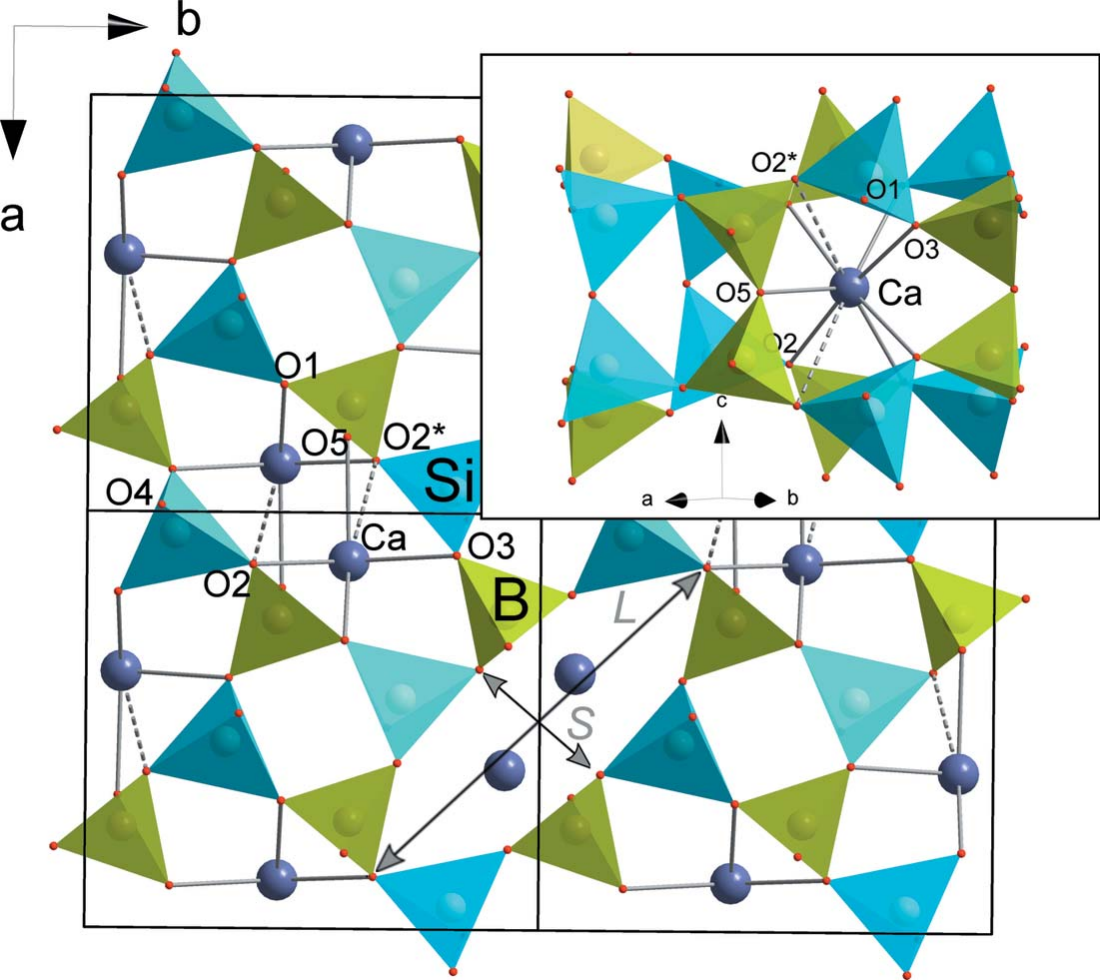
The crystal structure of danburite under ambient conditions, viewed along the *c* axis. Green and blue tetrahedra represent BO_4_ and SiO_4_, respectively. The Ca atoms are shown in dark blue. The inset shows the coordination of a Ca atom, where the dotted lines correspond to bond lengths exceeding 3 Å.

Under ambient conditions danburite, CaB_2_Si_2_O_8_, is orthorhombic in the space group *Pnam* and the unit cell is *a* = 8.038 (3), *b* = 8.752 (5) and *c* = 7.730 (3) Å (Phillips *et al.*, 1974 ▸). The asymmetric unit of danburite contains two tetrahedrally coordinated cations *T* (Si and B) and five O atoms (Fig. 1 ▸). Polymerization of Si_2_O_7_ and B_2_O_7_ structural units by common sharing of O atoms results in the formation of a tetrahedral framework, with channels outlined by four- and eight-membered rings along the *c* axis (Fig. 1 ▸). The channels with eight-membered rings are occupied by Ca atoms in either seven-fold (for Ca—O bonds shorter than 3 Å) or nine-fold coordination (taking into account two Ca—O bonds of 3.02 Å). The eight-membered rings consisting of Ca atoms are elliptically elongated. The elongation can be measured in terms of the *L*/*S* ratio between the longest (*L*) and shortest (*S*) diagonals, and it is equal to 1.33 under ambient conditions.

### 3.2. High-pressure phase transitions of danburite   

The high-pressure behaviour of danburite was investigated up to 36 GPa by synchrotron-based single-crystal X-ray diffraction (experiments 1 and 2) and by IR and Raman spectroscopy. The transformation route was found to be sensitive to hydro­static conditions within the sample chamber (see *Materials and methods* section[Sec sec2] for details and Fig. S1). The structural refinements performed in experiment 1 (Tables S1 and S2) are used for the following crystal chemical discussion, while the outlined transformation route was followed in experiment 2.

**Figure 2:**
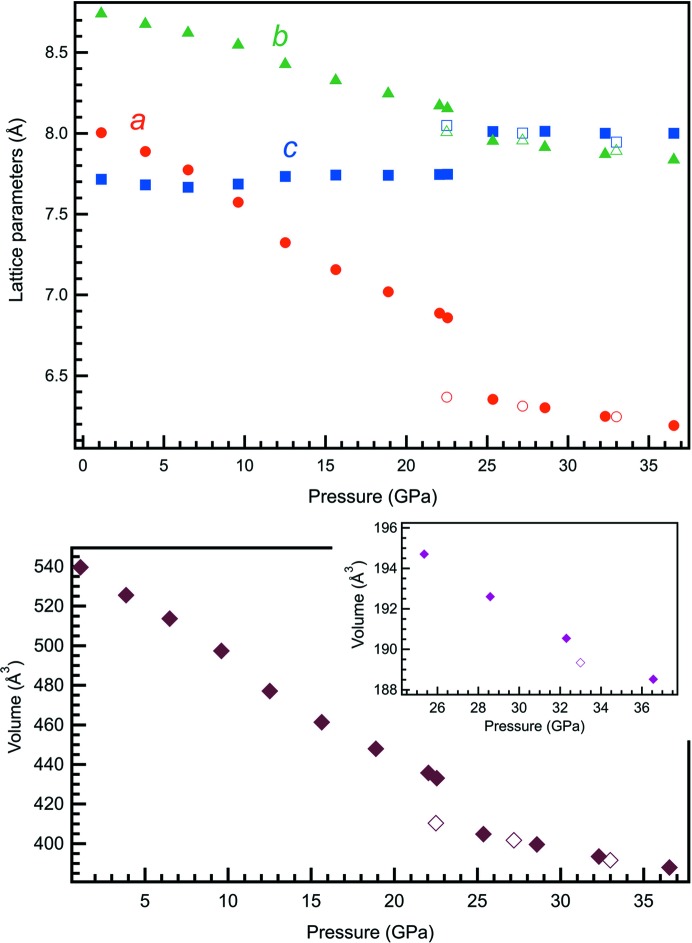
The evolution of the unit-cell parameters of danburite along the compression. The inset shows the dependence of the volume of the 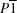 phase (danburite-III) on the pressure increase. Filled and open symbols correspond to experiments 1 and 2, respectively. The errors are smaller than the size of the symbols.

Below 6.5 GPa, continuous contraction of the unit-cell parameters (Fig. 2 ▸) and atomic bonds (Fig. S2) is observed, in agreement with the previous high-pressure diffraction study of danburite up to 4.6 GPa (Hackwell & Angel, 1992 ▸). The unit cell undergoes anisotropic compression, with the *a* axis displaying the highest compressibility and the *c* axis the lowest (Fig. S3). The stiffness of the *c* axis is governed by the low compressibility of the Si—O bonds through bridging O atoms in the Si_2_O_7_ ditetrahedral groups. In agreement with the earlier report, the compression of the material up to ∼6.5 GPa is controlled by changes in the *T*—O—*T* angles of the tetrahedral framework (Hackwell & Angel, 1992 ▸).

Unexpected behaviour of the crystal structure is observed above 6.5 GPa. While the *a* and *b* axes continue to decrease, the *c* axis reveals an anomalous increase (Figs. 2 ▸ and S3). Bulk moduli obtained by fitting of the *P*–*V* data with the Murnaghan equation of state below 6.5 GPa and between 6.5 and 23 GPa are 94.1 (4) and 33 (1) GPa, respectively (Table S3) (Angel, 2000 ▸). The difference in bulk modulus of almost three times indicates significant softening of the structure above ∼6.5 GPa. Anomalous behaviour is also observed for certain bond lengths (Fig. S2). Si and B atoms are displaced out of the centres of the *T*O_4_ tetrahedra. The deviation of the SiO_4_ and BO_4_ units from the ideal tetrahedral geometry at ∼10 GPa is clearly visible on plots showing the quadratic elongation (QE) and bond angular variation (BAV) parameters as a function of pressure (Fig. S4) (Robinson *et al.*, 1971 ▸).

**Figure 3:**
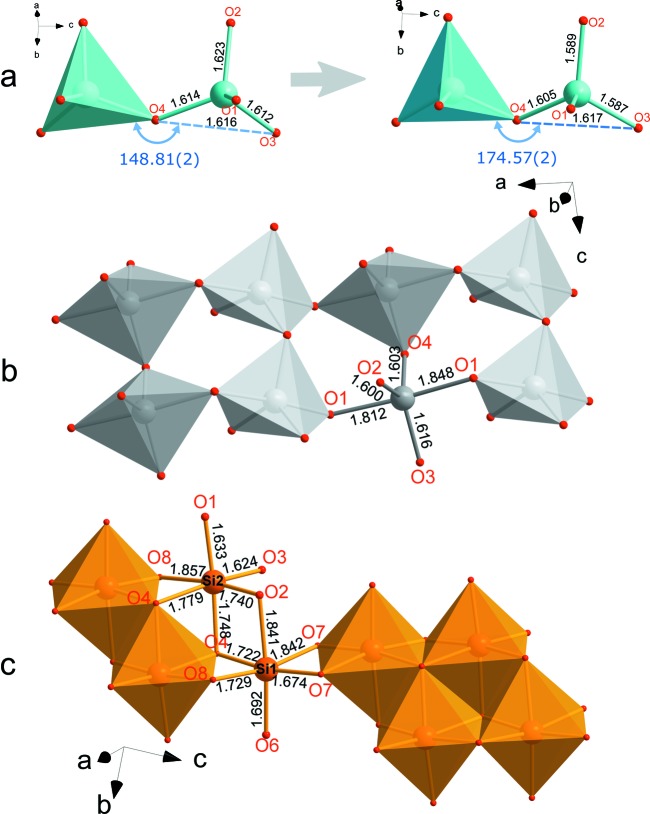
The evolution of silicon-based structural units along the high-pressure treatment of danburite. (*a*) Distortion of an Si_2_O_7_ ditetrahedral group from 1.1 (1) to 22.6 (1) GPa. Note the respective increase in the O3—O4—O3 angle from 148.81 (2) to 174.57 (2)° that causes the anomalous enlargement of the *c* axis along the compression. (*b*) A chain built of SiO_5_ trigonal bipyramids in the structure of danburite-II (*Pnam*) at 25.4 (1) GPa. (*c*) A chain built of edge-sharing SiO_6_ octahedra in danburite-III (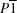) at 25.4 (1) GPa. The Si—O bond distances are given in ångströms.

Under compression to about 23 GPa the danburite framework undergoes severe distortion: the eight-membered rings become strongly elliptically distorted (the *L*/*S* ratio increases by almost three times from 1.33 to 3.27) and the *T*—O—*T* angles decrease from ∼126–138° to ∼118–126°. This last feature is also reflected in the evolution of the Raman band assigned for the Si—O—Si symmetric bending mode of the Si_2_O_7_ units (Garbev *et al.*, 2007 ▸): it shifts continuously from ∼615 cm^−1^ at 1.4 (1) GPa to ∼663 cm^−1^ at 22.7 (1) GPa (Fig. S5). Framework distortion is particularly pronounced in the rotation of tetrahedra in the *T*_2_O_7_ groups (Fig. 3 ▸ *a*) which is responsible for the anomalous increase in the *c* parameter with pressure. Up to ∼23 GPa, neither discontinuity in volume nor changes in symmetry of the structure and Wyckoff positions of individual atoms are observed. Still, the Raman and IR spectra in this pressure range are significantly affected by the progressive distortion of the coordination environment of the cations, indicating that not only does the compressive mechanism change with accompanying structure softening and the material become softer, but the vibrational properties of danburite are very different below and above ∼10 GPa. Thus, the IR band at ∼917 cm^−1^ at 2.5 (1) GPa, which gradually shifts to 940 cm^−1^ at 9.2 (1) GPa and then successively moves back to lower frequencies [∼917 cm^−1^ at ∼21.2 (1) GPa], is most probably due to vibrations of the B—O5 and/or B—O3 bonds (Fig. S6).

Above ∼23 GPa the crystal structure of danburite undergoes a phase transition that is reflected in the abrupt change in its vibrational spectra (Figs. S5 and S6) and unit-cell parameters (Figs. 2 ▸ and S3). The high-pressure phase (danburite-II) preserves the orthorhombic (*Pnam*) symmetry while the structural change is displacive and is induced by a shift of the Si atoms along the *a* axis in such a way that an extra O1* atom of an adjacent tetrahedron, across the eight-membered ring, joins its coordination sphere (Fig. S7*a*), so that the Si co­ordination becomes fivefold. The Si—O—Si symmetric bending mode of the Si_2_O_7_ ditetrahedral unit of danburite is not visible in the Raman spectrum above 25 GPa, in agreement with the absence of tetrahedral silicon above this pressure. At 25.4 (1) GPa, the coordination polyhedron of Si has trigonal–bipyramidal geometry (Fig. 3 ▸ *b*) with two long (1.81–1.85 Å) apical and three short (1.60–1.62 Å; Table S2) equatorial Si—O bonds. The O—Si—O apical bond angle is 7.3° away from the 180° angle required for a regular trigonal bipyramid. More detailed inspection of the SiO_5_ geometry can be performed through the Liebau approach (Liebau, 1984 ▸), where the mean 〈O_eq_—Si—O_eq_〉, 〈O_eq_—Si—O_ap_〉 and 〈O_eq_—Si—O_ap_′〉 angles are considered (O_eq_ are equatorial O atoms, while O_ap_ and O_ap_′ are the two apical O atoms with shorter and longer Si—O bonds, respectively). These angles in the structure of danburite-II are 119.9, 88.6 and 91.4° respectively, indicating that the SiO_5_ polyhedron is exceptionally close to the values of 120° and 90° characteristic for an ideal trigonal bipyramid. The SiO_5_ trigonal bipyramids share common vertices to form chains running along the *a* axis. The connection along the *c* axis through bridging O atoms is preserved and leads to the formation of double [Si_2_O_7_]_∞_ chains (Fig. 3 ▸ *b*). To the best of our knowledge, danburite-II is the first example of a high-pressure phase possessing silicon in a fivefold coordination only. Moreover, the geometry of a trigonal bipyramid has not previously been reported before for silicates, although it is known in metal–organic compounds (Rappoport & Apeloig, 1998 ▸; Baus *et al.*, 2013 ▸; Wagler *et al.*, 2014 ▸; Mück *et al.*, 2016 ▸).

Upon compression above 32 GPa, the appearance of a new high-pressure phase, co-existing with danburite-II (Fig. S1), is observed. The structure of this new phase, named danburite-III, was solved and refined in the 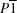 space group (Table S1, and Figs. 3 ▸ *c* and S7*b*). The crystal structure contains two crystallographically independent Si, two B, one Ca and eight O sites. The *Pnam* → 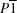 transition is reconstructive in character and involves breaking of chemical bonds and re-formation of the bonding network within Si-based chains. Across the transition, the chain composed of corner-sharing SiO_5_ trigonal bipyramids transforms into chains of edge-sharing SiO_6_ octahedra (Fig. 3 ▸ *c*). The bond-length distributions are similar for both Si sites and are in the range 1.62–1.86 Å (Table S2). We are not aware of any other example of this kind of topology consisting of octahedral chains with six-coordinated Si atoms in silicates.

## 4. Discussion   

**Figure 4:**
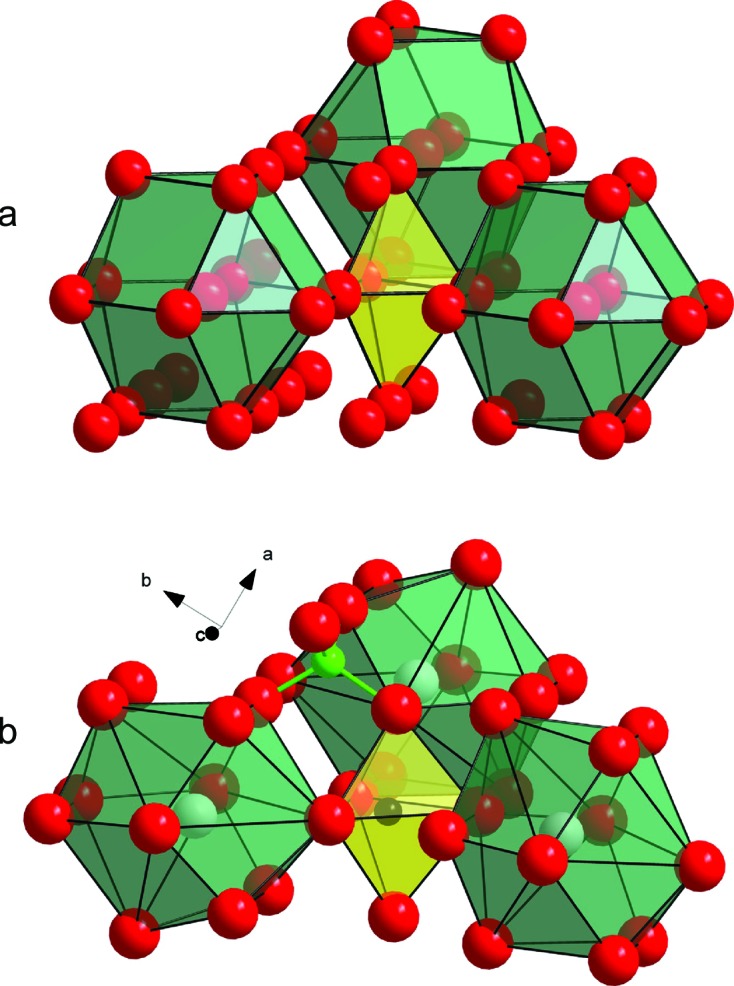
Drawing parallels between the danburite-II crystal structure and an ideal h.c.p. structure. (*a*) A view of an h.c.p. structure based on hexagonal cubo­octahedra (shown in green). A void with trigonal–bipyramidal geometry is shown in yellow. (*b*) A representation of the crystal structure of danburite-II based on a distorted h.c.p. structure made of Ca and O atoms. CaO_11_ polyhedra (defective hexagonal cubo­octahedra) are shown in green. The void with trigonal–bipyramidal geometry is occupied by an Si atom (shown in black). The bright-green sphere towards the top centre of panel (*b*) represents the B atom occupying a tetrahedral void.

Pentacoordinated silicon is extremely rare in inorganic compounds, and the reason why SiO_4_ tetrahedra transform directly into SiO_6_ octahedra without transitional SiO_5_ co­ordination is not clear. Liebau (1984 ▸) explained the absence of [SiO_5_] groups in terms of the ionic character of chemical bonding in silicates. The difference in electronegativities between Si and O ions (Δχ) is relatively high at 1.76; for comparison, the Δχ values for Si—N and Si—C pairs are 1.33 and 0.76, respectively, enabling the occurrence of Si*X*_5_ groups in organosilicon compounds. Ionic oxide compounds are known for their tendency of forming a close-packed arrangement of oxygen atoms, with cations filling up the voids between the anions. Many silicates under ambient conditions can be described as distorted derivatives of cubic or hexagonal close-packed (c.c.p. and h.c.p., respectively) layer structures (Liebau, 1985 ▸; Lima-de-Faria, 1994 ▸; Krivovichev, 2009 ▸; Thompson & Downs, 2003 ▸). The application of high pressure forces a rearrangement of the structural units so as to occupy the space most efficiently, *i.e.* the oxygen sublattice evolves towards an ideal close packing. In close packings there are only tetrahedral and octahedral voids, which explains the common presence of four- and six-coordinated silicon (Liebau, 1984 ▸). However, h.c.p. arrays may be considered as containing pentacoordinated voids with a trigonal–bipyramidal geometry. These voids are formed by three spheres in a layer and two spheres above and below the layer, *i.e.* they are formed by merging two tetrahedral voids (Figs. 4 ▸ *a* and S8). However, the size of the void within the plane of the layer is so small that no cations can fit inside. For example, assuming an ideal close packing of O^2−^ anions with an ionic radius of 1.32 Å, the cation would have to have a radius of ∼0.2 Å. It is noteworthy that filling of pentacoordinated voids has been repeatedly reported for metallic and intermetallic compounds (Isaeva *et al.*, 2010 ▸; Larsson *et al.*, 1995 ▸), as well as for hexagonal *M*-type ferrites (Townes *et al.*, 1967 ▸; Kreber & Gonser, 1976 ▸; Albanese *et al.*, 1981 ▸). The possible presence of Si in a trigonal–bipyramidal void has also been discussed for the CaAl_4_Si_2_O_11_ phase with a close-packed structure based upon the hexagonal barium ferrite model (Gautron *et al.*, 1999 ▸). Our work demonstrates that densification of crystal structures may involve an intermediate stage of atomic arrangement with occupied trigonal–bipyramidal voids.

**Figure 5:**
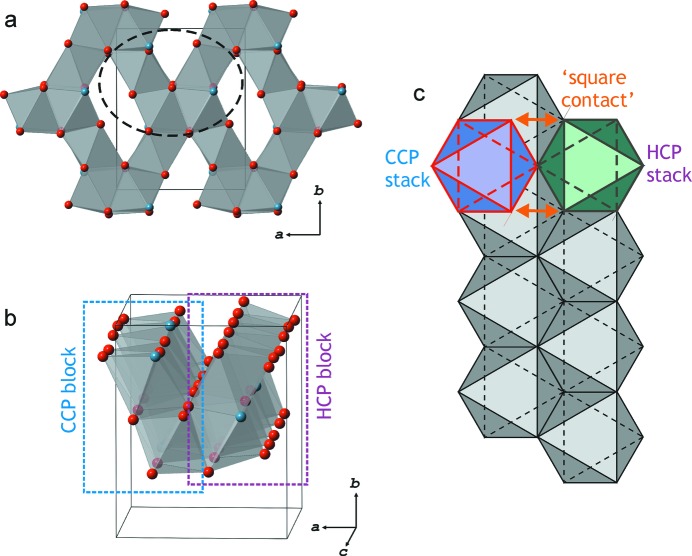
Representation of the crystal structure of danburite-II on the basis of the close-packing approach. (*a*) The arrangement of octahedral voids in the packing of O (red) and Ca (blue) atoms in danburite-II, featuring chains of edge-sharing octahedral voids parallel to the *c* axis. The area outlined by the dashed line is magnified in panel (*b*). (*b*) Four chains of edge-sharing octahedral voids making up adjacent c.c.p. and h.c.p. blocks. (*c*) A scheme showing the origin of the ‘square contact’ between the c.c.p. and h.c.p. blocks through the cover of underlying double chains of octahedra by octahedra in either a c.c.p. (lilac octahedron) or h.c.p. (green octahedron) fashion.

Under ambient conditions danburite possesses a relatively open structure, which has no direct relation to the close-packed arrangements of the O atoms. In contrast, danburite-III is dense and its structure is based upon the c.c.p. arrangement formed by both Ca and O atoms (Fig. S9). The close-packed layers are coplanar with the (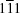) plane, with Si occupying 2/9 of all octahedral voids and B occupying 2/18 of all tetrahedral voids. The structure of danburite-II can be described as having elements of distorted c.c.p and h.c.p. close packings. It contains ribbons of close-packed O and Ca atoms parallel to the (010) plane and extended along the *c* axis. Fig. 5 ▸(*a*) shows the arrangement of the octahedral voids, organized into chains running along [001] in the structure, whereas Figs. 5 ▸(*b*) and 5 ▸(*c*) specify the deviations of the packing from both h.c.p. and c.c.p. The double chain of edge-sharing octahedral voids (shown in grey in Fig. 5 ▸ *c*) is covered by two adjacent chains of octahedral voids, one according to c.c.p. stacking (blue octahedra) and the other according to h.c.p. stacking (green octahedra). As a result, a square contact appears on the contact of the blue and green octahedra, which is not allowed in an ideal close-packed structure. Deviation from a close-packed geometry is manifested in the coordination number of Ca (11, instead of 12 in the ideal situation; Belov, 1941 ▸; Giacovazzo *et al.*, 2002 ▸). Nevertheless, the presence of close-packed arrangements in danburite-II compared with danburite-I shows that, under increasing pressure, the structure evolves towards a close-packed architecture, which is realised in danburite-III. In danburite-II, trigonal–bipyramidal voids within close-packed structure regions are occupied by Si, whereas tetrahedral voids are occupied by B (Fig. 4*b* ▸). The danburite-II → danburite-III transition involves [h.c.p. + c.c.p.] → c.c.p. transformation and therefore has a distinct reconstructive character.

The mechanism of the pressure-induced phase transition invoked by our experimental work on danburite can be applied to explain the observation of silicon in a (4+1) co­ordination in the high-pressure modification of Mg–Fe orthoenstatite (OEn) with the chemical formula (Mg_0.900_Fe_0.088_Ca_0.003_Mn_0.003_)(Al_0.004_Si_0.999_)O_3_ (Finkelstein *et al.*, 2015 ▸). Under ambient conditions, the oxygen sublattice of OEn is described by a distorted *ABCBACBC* sequence (Thompson & Downs, 2003 ▸). Compression to ∼30 GPa results in a transition to α-postOEn, with the O atoms forming a nearly ideal *ABCBACBC* pattern; further compression leads to the ∼40 GPa transition to β-postOEn with the …*AB*… stacking sequence (Fig. S10) (Isaeva *et al.*, 2010 ▸; Larsson *et al.*, 1995 ▸). The shifts in the packing of the O atoms towards an h.c.p. structure result in the transformation of the tetrahedral voids into octahedral ones. The fivefold coordination of Si forms due to the asynchronization of two processes: formation of octahedral voids and movement of Si atoms into the centres of these voids. Therefore, Si moves from the equatorial plane of the octahedron and its coordination polyhedron can be considered as a distorted square pyramid (Fig. S10*b*). The same mechanism may be responsible for the formation of the very first known silicate with an SiO_5_ group, triclinic CaSi_2_O_5_: the phase is found only in a very limited pressure range and, upon the transition at 0.2 GPa, SiO_5_ square pyramids transform into octahedra (Angel *et al.*, 1996 ▸; Angel, 1997 ▸). By analogy with CaSi_2_O_5_, the filling of trigonal voids in CaAl_4_Si_2_O_11_ was proposed to be pressure-dependent and the transition from four- to five-coordinated silicon was expected to occur upon compression (Gautron *et al.*, 1999 ▸).

Our results on the phase transitions of danburite and comparisons with other known examples of silicates with pentacoordinated silicon suggest that SiO_5_ groups may be not as rare as previously thought and may form as intermediate configurations upon (*a*) transformation of silicates with relatively open structures into phases based upon close packing of oxygen (or oxygen and large cations), and (*b*) transformations of close-packed structures that involve transition between octahedral and tetrahedral voids due to shifts of close-packed layers. It is very likely that the presence of boron as the second framework-forming cation plays an important role in the discovered transformation route of danburite. Further studies are needed to illuminate how the interplay between the two cations influences the high-pressure structural behaviour of silicates.

## Supplementary Material

Crystal structure: contains datablock(s) danburite_1.1GPa, danburite_22.6GPa, danburite_25.4GPa, danburite-III_25.4GPa, danburite-IV_32.3GPa. DOI: 10.1107/S2052252517010612/lt5006sup1.cif

Structure factors: contains datablock(s) danburite_22.6Gpa. DOI: 10.1107/S2052252517010612/lt5006danburite_22.6Gpasup2.hkl

Structure factors: contains datablock(s) danburite_25.4GPa. DOI: 10.1107/S2052252517010612/lt5006danburite_25.4GPasup3.hkl

Structure factors: contains datablock(s) danburite-III_25.4GPa. DOI: 10.1107/S2052252517010612/lt5006danburite-III_25.4GPasup4.hkl

Structure factors: contains datablock(s) danbburite-IV_32.3GPa. DOI: 10.1107/S2052252517010612/lt5006danburite-IV_32.3GPasup5.hkl

Supplementary tables and figures. DOI: 10.1107/S2052252517010612/lt5006sup6.pdf

CCDC references: 1562973, 1562974, 1562975, 1562976

## References

[bb1] Agilent (2012). *CrysAlisPRO.* Agilent Technologies, Yarnton, Oxfordshire, England.

[bb2] Albanese, G., Deriu, A., Lucchini, E. & Slokar, G. (1981). *Appl. Phys. A*, **26**, 45–50.

[bb3] Alberti, A., Sacerdoti, M., Quartieri, S. & Vezzalini, G. (1999). *Phys. Chem. Miner.* **26**, 181–186.

[bb4] Angel, R. J. (1997). *Am. Miner.* **82**, 836–839.

[bb5] Angel, R. J. (2000). *Rev. Mineral. Geochem.* **41**, 35–59.

[bb6] Angel, R. J., Gonzalez-Platas, J. & Alvaro, M. (2014). *Z. Kristallogr.* **229**, 405–419.

[bb7] Angel, R. J., Ross, N. L., Seifert, F. & Fliervoet, T. F. (1996). *Nature*, **384**, 441–444.

[bb8] Baus, J. A., Burschka, C., Bertermann, R., Guerra, C. F., Bickelhaupt, F. M. & Tacke, R. (2013). *Inorg. Chem.* **52**, 10664–10676. 10.1021/ic401698a 24011307

[bb9] Belov, N. B. (1941). *Structure of Ionic Crystals and Metallic Phases*, p. 144. Moscow: Academy of Sciences USSR Publishers.

[bb10] Best, S. P., Clark, R. J. H., Hayward, C. L. & Withnall, R. (1994). *J. Raman Spectrosc.* **25**, 557–563.

[bb11] Finger, R. M. & Hazen, L. W. (2000). *Rev. Mineral. Geochem.* **41**, 123–155.

[bb12] Finkelstein, G. J., Dera, P. K. & Duffy, T. S. (2015). *Phys. Earth Planet. Inter.* **244**, 78–86.

[bb13] Garbev, K., Stemmermann, P., Black, L., Breen, C., Yarwood, C. & Gasharova, B. (2007). *J. Am. Ceram. Soc.* **90**, 900–907.

[bb14] Gautron, L., Angel, R. J. & Miletich, R. (1999). *Phys. Chem. Miner.* **27**, 47–51.

[bb15] Giacovazzo, C., Monaco, H. L., Viterbo, D., Scordari, F., Gilli, G., Zanotti, G. & Catti, M. (2002). *Fundamentals of Crystallography*, pp. 825. New York: Oxford University Press.

[bb16] Goryainov, S. V. (2016). *J. Raman Spectrosc.* **47**, 984–992.

[bb17] Hackwell, T. H. & Angel, R. J. (1992). *Eur. J. Mineral.* **4**, 1221–1228.

[bb18] Isaeva, A. A., Makarevich, O. N., Kuznetsov, A. N., Doert, T., Abakumov, A. M. & Van Tendeloo, G. (2010). *Eur. J. Inorg. Chem.* **2010**, 1395–1404.

[bb19] Kantor, I., Prakapenka, V., Kantor, A., Dera, P., Kurnosov, A., Sinogeikin, S., Dubrovinskaia, N. & Dubrovinsky, L. (2012). *Rev. Sci. Instrum.* **83**, 125102. 10.1063/1.4768541 23278021

[bb20] Karas, O. A., Pakhomova, V. A., Karabtsov, A. A. & Kononov, V. V. (2008). *Proceedings of XIII International Conference on Thermobarogeochemistry and IV APIFIS Symposium*, 22–25 September 2008, Moscow, Russia, Vol. 2, pp. 45–48. Moscow: INEG RAN.

[bb21] Kreber, U. & Gonser, E. (1976). *Appl. Phys.* **10**, 175–180.

[bb22] Krivovichev, S. V. (2009). *Structural Crystallography of Inorganic Oxysalts*, Vol. 22, p. 328. Oxford University Press.

[bb23] Kurnosov, A., Kantor, I., Boffa-Ballaran, T., Lindhardt, S., Dubrovinsky, L., Kuznetsov, A. & Zehnder, B. H. (2008). *Rev. Sci. Instrum.* **79**, 045110. 10.1063/1.2902506 18447555

[bb24] Larsson, A.-K., Stenberg, L., Lidin, S. & Hase, T. (1995). *Acta Chem. Scand.* **49**, 800–802.

[bb25] Liebau, F. (1984). *Inorg. Chim. Acta*, **89**, 1–7.

[bb26] Liebau, F. (1985). *Structural Chemistry of Silicates*, p. 354. Heidelberg: Springer-Verlag.

[bb27] Lima-de-Faria, J. (1994). *Structural Mineralogy: An Introduction*, p. 346. Dordrecht: Springer Netherlands.

[bb28] Mao, H. K., Xu, J. & Bell, P. M. (1986). *J. Geophys. Res.* **91**, 4673–4676.

[bb29] Mück, F. M., Förster, B., Baus, J. A., Nutz, M., Burschka, C., Bertermann, R. & Tacke, R. (2016). *Eur. J. Inorg. Chem.* 3246–3252.

[bb30] Pauling, L. (1929). *J. Am. Chem. Soc.* **51**, 1010–1026.

[bb31] Phillips, M. W., Gibbs, G. V. & Ribbe, P. H. (1974). *Am. Mineral.* **59**, 79–85.

[bb32] Prewitt, C. T. & Downs, R. T. (1998). *Rev. Mineral. Geochem.* **37**, 283–317.

[bb33] Rappoport, Z. & Apeloig, Y. (1998). *The Chemistry of Organic Silicon Compounds*, Vol. 2, p. 2830. Chichester: John Wiley & Sons Ltd.

[bb34] Robinson, K., Gibbs, G. V. & Ribbe, P. H. (1971). *Science*, **172**, 567–570. 10.1126/science.172.3983.567 17802221

[bb35] Sheldrick, G. M. (2015). *Acta Cryst.* C**71**, 3–8.

[bb36] Thompson, R. M. & Downs, R. T. (2003). *Am. Mineral.* **88**, 653–666.

[bb37] Townes, W. D., Fang, J. H. & Perrotta, A. J. (1967). *Z. Kristallogr.* **125**, 437–449.

[bb38] Wagler, J., Böhme, U. & Kroke, E. (2014). *Struct. Bond.* **155**, 29–106.

