# Supplementary material for: A closer look into close packing: pentacoordinated silicon in a high-pressure polymorph of danburite
Source: IUCrJ. 2017 Aug 10;4(Pt 5):671–7. doi: 10.1107/S2052252517010612 (PMC5619858; doi:10.1107/S2052252517010612)
Supplement: Supplementary file 6 [file m-04-00671-sup6.pdf]

# IUCrJ

**Volume 4 (2017)**


**Supporting information for article:**

## A closer look at close packing: pentacoordinated silicon in a high-pressure polymorph of danburite

**A. Pakhomova, E. Bykova, M. Bykov, K. Glazyrin, B. Gasharova, H.-P. Liermann, M. Mezouar, L. Gorelova, S. Krivovichev and L. Dubrovinsky**

**Table S1**. Crystallographic data and refinement parameters for danburite polymorphs

| Crystal data | 1.1(1) GPa | 22.6(1) GPa | 25.4(1) GPa | 25.4(1) Pa | 32.3(1) GPa |
|---|---|---|---|---|---|
| | Danburite | | Danburite-II | Danburite-III | Danburite-IV |
| Space group | *Pnam* | *Pnam* | *Pnam* | *P*-1 | $P2_1/c$ |
| *a*, Å | 8.0035(2) | 6.8576(3) | 6.3537(5) | 5.479(5) | 7.9989(17) |
| *b*, Å | 8.7386(3) | 8.1526(3) | 7.9518(3) | 5.532(2) | 7.8697(14) |
| *c*, Å | 7.7147(2) | 7.7457(2) | 8.0112(3) | 6.681(6) | 6.249(3) |
| α, ° | 90 | 90 | 90 | 91.74(5) | 90 |
| β, ° | 90 | 90 | 90 | 104.57(8) | 89.75(3) |
| γ, ° | 90 | 90 | 90 | 95.59(5) | 90 |
| Volume, Å$^3$ | 539.56(3) | 433.04(3) | 404.75(4) | 194.7(3) | 393.4(2) |
| *Z* | 4 | 4 | 4 | 2 | 4 |
| Data collection | | | | | |
| Wavelength, Å | 0.29024 | 0.29024 | 0.29056 | 0.29056 | 0.29056 |
| Max. $\theta$° | 17.189 | 16.875 | 16.730 | 11.151 | 10.458 |
| Index ranges | $-14\le h\le 13$ | $-10\le h\le 11$ | $-10\le h\le 9$ | $-6\le h\le 6$ | $-9\le h\le 9$ |
| | $-13\le k\le 12$ | $-13\le k\le 12$ | $-13\le k\le 12$ | $-7\le k\le 7$ | $-9\le k\le 9$ |
| | $-13\le l\le 14$ | $-13\le l\le 15$ | $-15\le l\le 14$ | $-8\le l\le 7$ | $-6\le l\le 6$ |
| No.meas.refl. | 3169 | 2397 | 2163 | 1042 | 940 |
| No.uniq.refl. | 1322 | 977 | 891 | 591 | 524 |
| No. obs.refl | 1241 | 926 | 742 | 397 | 408 |

| | | | | | |
|---|---|---|---|---|---|
| ($I > 2\sigma(I)$) | | | | | |
| *Refinement of the structure* | | | | | |
| No.of variables | 64 | 65 | 65 | 68 | 68 |
| $R_{int}$ | 0.0251 | 0.0191 | 0.0239 | 0.0545 | 0.0536 |
| $R_{\sigma}$ | 0.0303 | 0.0229 | 0.0291 | 0.0934 | 0.0587 |
| $R_1$, $I > 2\sigma(I)$ | 0.0219 | 0.0262 | 0.0423 | 0.1154 | 0.0982 |
| $R_1$, all data | 0.0235 | 0.0293 | 0.0596 | 0.0887 | 0.1254 |
| w$R_2$, $I > 2\sigma(I)$ | 0.0651 | 0.0698 | 0.1256 | 0.2368 | 0.2944 |
| w$R_2$, all data | 0.0663 | 0.0723 | 0.1783 | 0.2714 | 0.3099 |
| GooF | 1.218 | 1.190 | 1.321 | 1.120 | 1.178 |

**Table S2**. Bond distances and polyhedral parameters in danburite, $CaB_2(SiO_4)_2$

| Pressure | **1.1(1) GPa** | **22.6(1) GPa** | | **25.4(1) GPa** | | **32.3(1) GPa** | | | | **25.4(1) GPa** | | |
|---|---|---|---|---|---|---|---|---|---|---|---|---|
| Phase | **Danburite** | | | **Danburite-II** | | **Danburite-IV** | | | | **Danburite-III** | | |
| | ***Pnam*** | | | ***Pnam*** | | ***P*2₁/*c*** | | | | ***P*-1** | | |
| CN(Si) | ***4*** | | | ***5*** | | ***5*** | | | | ***6*** | | |
| *$SiO_n$ polyhedra* | | | | | | | | | | | | |
| | ***$SiO_4$*** | | | ***$SiO_5$*** | | ***Si1O₅*** | | ***Si2O₅*** | | ***Si1O₆*** | | ***Si2O₆*** |
| **Si-O3** | 1.6119(7) | 1.5875(9) | | 1.6157(19) | **Si1-O4** | 1.59(1) | **Si2-O4** | 1.603(9) | **Si1-O3** | 1.625(9) | **Si2-O7** | 1.672(9) |
| **Si-O4** | 1.6145(4) | 1.6044(7) | | 1.6030(15) | **Si1-O8** | 1.603(9) | **Si2-O7** | 1.607(9) | **Si1-O1** | 1.631(7) | **Si2-O6** | 1.696(8) |
| **Si-O1** | 1.6162(7) | 1.617(1) | | 1.812(2) | **Si1-O2** | 1.621(8) | **Si2-O1** | 1.623(9) | **Si1-O2** | 1.744(9) | **Si2-O4** | 1.724(9) |
| **Si-O2** | 1.6232(7) | 1.5894(8) | | 1.600(2) | **Si1-O6** | 1.79(1) | **Si2-O5** | 1.787(11) | **Si1-O4** | 1.745(7) | **Si2-O8** | 1.728(9) |
| | | | **Si-O1*** | 1.848(2) | **Si1-O6** | 1.81(1) | **Si2-O5** | 1.814(11) | **Si1-O4** | 1.773(9) | **Si2-O2** | 1.837(7) |
| | | | | | | | | | **Si1-O8** | 1.861(9) | **Si2-O7** | 1.839(9) |
| **<Si-O>** | 1.6165 | 1.5995 | | 1.696 | | 1.6837 | | 1.6872 | | 1.7299 | | 1.7490 |
| **Volume** | 2.165 | 2.066 | | 4.0737 | | 4.0005 | | 4.0340 | | 6.814 | | 7.024 |
| *$BO_4$ tetrahedra* | | | | | | | | | | | | |
| | ***$BO_4$*** | | | | | ***B1O₄*** | | ***B2O₄*** | | ***B1O₄*** | | ***B2O₄*** |
| **B-O5** | 1.450(1) | 1.446(1) | | 1.456(3) | **B1-O7** | 1.44(1) | **B1-O8** | 1.42(2) | **B1-O3** | 1.398(16) | **B2-O2** | 1.426(15) |
| **B-O3** | 1.462(1) | 1.473(2) | | 1.471(4) | **B1-O1** | 1.44(2) | **B1-O2** | 1.46(2) | **B1-O5** | 1.445(16) | **B2-O5** | 1.456(13) |
| **B-O1** | 1.480(1) | 1.429(1) | | 1.461(3) | **B1-O5** | 1.45(2) | **B1-O6** | 1.46(2) | **B1-O8** | 1.476(14) | **B2-O6** | 1.468(17) |
| **B-O2** | 1.499(1) | 1.457(2) | | 1.444(3) | **B1-O3** | 1.46 (2) | **B1-O3** | 1.46(2) | **B1-O7** | 1.577(13) | **B2-O1** | 1.509(12) |
| **<B-O>** | 1.473 | 1.451 | | 1.458 | | 1.4462 | | 1.4504 | | 1.4742 | | 1.464 |
| **Volume** | 1.629 | 1.556 | | 1.572 | | 1.5313 | | 1.5432 | | 1.6396 | | 1.6063 |

| | | | | *$CaO_n$ polyhedra* | | | | | | | |
|---|---|---|---|---|---|---|---|---|---|---|---|
| | ***$CaO_7$*** | ***$CaO_{11}$*** | ***$CaO_{11}$*** | ***$CaO_{11}$*** | | | | | | ***$CaO_{12}$*** | |
| **Ca-O5** | 2.3847(11) | 2.3218(15) | 2.354(3) | **Ca-O3** | 2.309(11) | **Ca-O3** | 2.468(9) | **Ca-O6** | 2.372(8) | **Ca-O2** | 2.515(8) |
| **Ca-O2 (x2)** | 2.4396(7) | 2.3614(8) | 2.3614(19) | **Ca-O8** | 2.329(8) | **Ca-O6** | 2.601(9) | **Ca-O5** | 2.404(7) | **Ca-O7** | 2.530(8) |
| **Ca-O3 (x2)** | 2.4575(7) | 2.5392(9) | 2.403(2) | **Ca-O7** | 2.339(9) | **Ca-O2** | 2.605(8) | **Ca-O8** | 2.431(8) | **Ca-O4** | 2.566(8) |
| **Ca-O1 (x2)** | 2.4815(7) | 2.4574(8) | 2.603(2) | **Ca-O1** | 2.355(9) | **Ca-O5** | 2.615(9) | **Ca-O6** | 2.447(8) | **Ca-O1** | 2.585(9) |
| **Ca-O2* (x2) ^** | 3.0173(7) | *3.2423(9)* | *3.407(3)* | **Ca-O2** | 2.358(11) | **Ca-O1** | 2.613(9) | **Ca-O1** | 2.467(7) | **Ca-O3** | 2.594(8) |
| **Ca-O4*** | *3.6127(7)* | 2.6152(12) | 2.405(3) | **Ca-O4** | 2.375(9) | | | **Ca-O3** | 2.504(8) | **Ca-O5** | 2.621(9) |
| **Ca-O5*** | *3.4788(7)* | 2.6336(12) | 2.516(3) | | | | | | | | |
| **Ca-O3*(x2)** | *4.626* | 2.8786(11) | 2.651(2) | | | | | | | | |
| <Ca-O> | 2.4595 /2.5990^ | 2.549 | 2.483 | | 2.4511 | | | | | 2.503 | |
| Volume | 18.774/ 28.1962^ | 36.083 | 33.791 | | 32.5420 | | | | | 37.030 | |

**Table S3.** Coefficients obtained by fitting the Murnaghan equation of state to the P-V data of danburite up to 23 GPa

| **Pressure range** | **$V_0$ (Å$^3$)** | **K (GPa)** | **Reference** |
|---|---|---|---|
| 0 - 7 GPa | 545.90(8) | 94.1(4) | present study |
| 7 - 23 GPa | 602(4) | 33(1) | present study |
| 0 - 4.5 GPa | 543.8(2) | 114(3) | Hackwell & Angel, 1974 |

* All calculations used K' fixed to 4.0 to provide a consistent basis for comparison between present and previously reported results. The observed difference in K in the low pressure region may indicate that the change in the compression mechanism of danburite has already occurred at 6.5 GPa. The lack of P-V data below 7 GPa (diffraction data was collected at three pressure points) prevented the precise estimation of critical pressure.

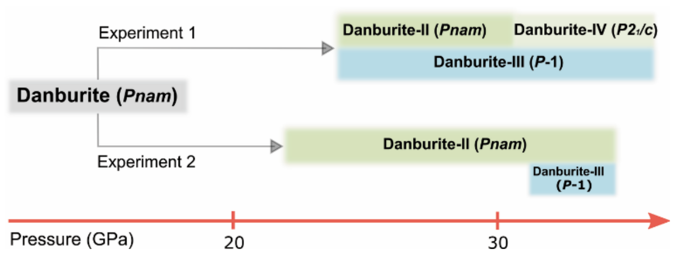


Fig. S1. The transformation routes of danburite in experiments 1 and 2.

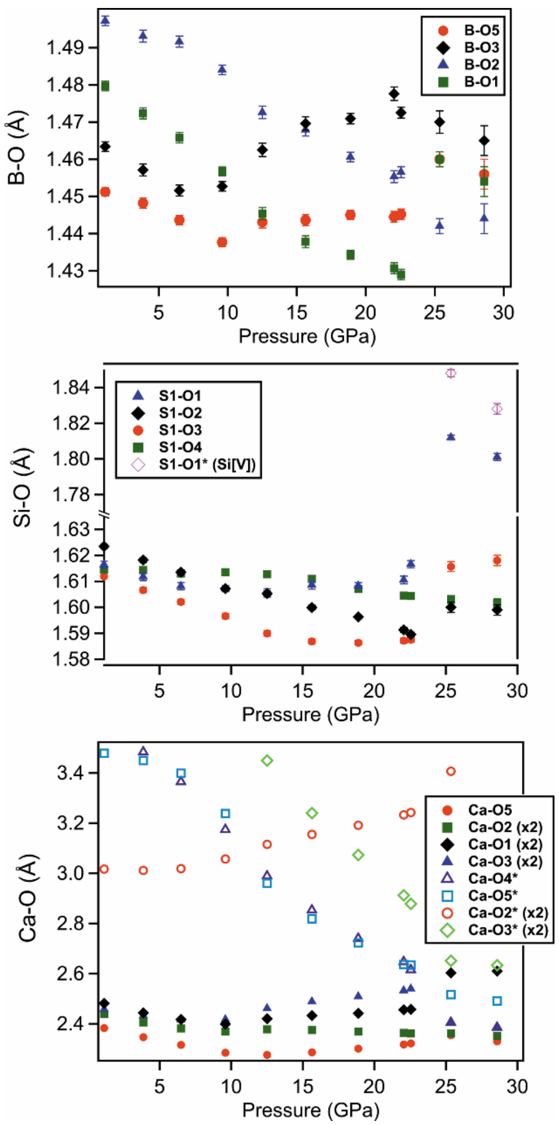


Fig. S2. High-pressure evolution of selected bond distances in *Pnam* phases of danburite.

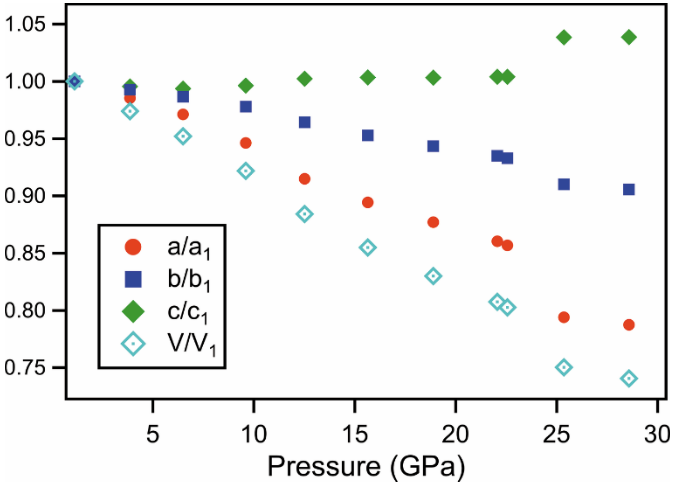


Fig. S3. Evolution of normalized unit-cell parameters of danburite and danburite-II. Data from Experiment 1 are presented.

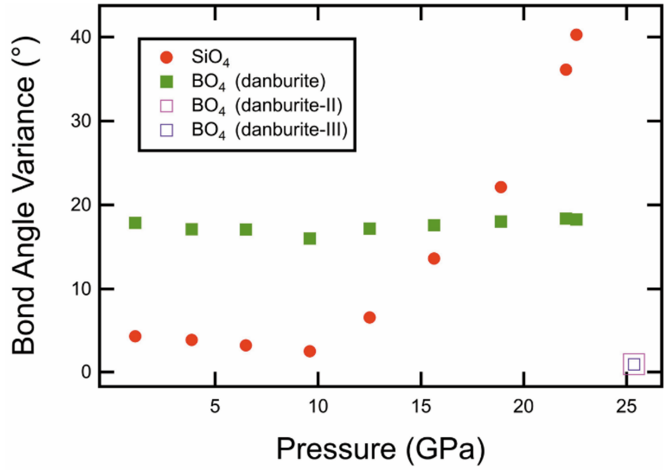


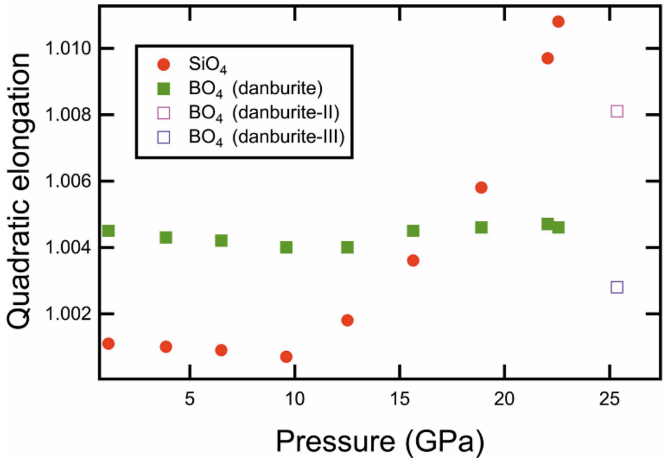


Fig. S4. The evolution of quadratic elongation (QE) and bond angle variance (BAV,°) of $SiO_4$ and $BO_4$ tetrahedra along the compression of danburite (Experiment 1). The parameters QE and BAV that show deviation of $TO_4$ polyhedra from geometry of ideal tetrahedron are defined as: QE = $\frac{1}{4}\sum_{i=1}^{4}\left(\frac{l_i}{l_0}\right)^2$ and BAV = $\sqrt{\frac{1}{5}\sum_{i=1}^{6}(\theta_i - 109.47)^2}$ where $l_0$ is a centre-to-vertex distance for ideal tetrahedron whose volume is equal to that of the distorted tetrahedron with bond lengths $l_i$ and bond angles $\theta_i$.

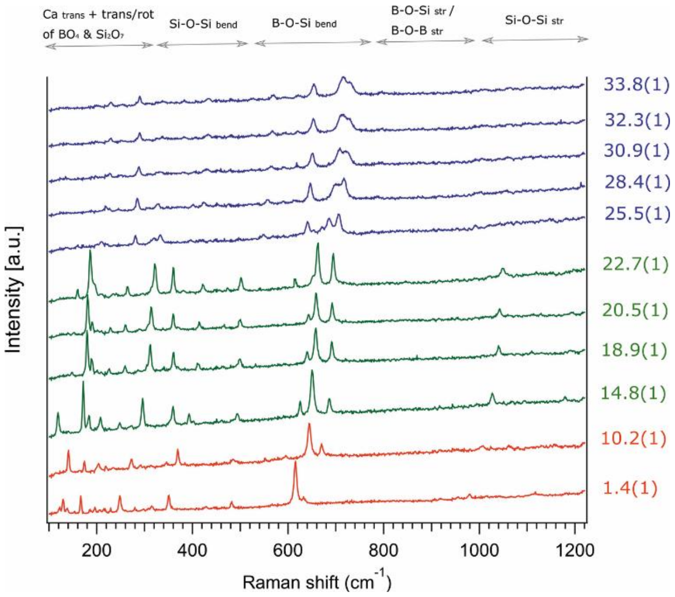


Fig. S5. Evolution of Raman spectra of danburite along the compression. The pressure is given in GPa. The peaks are assigned according to Best et al. (1994) (measurements performed under ambient conditions). Different colours correspond to the high-pressure phases with distinct vibration properties.

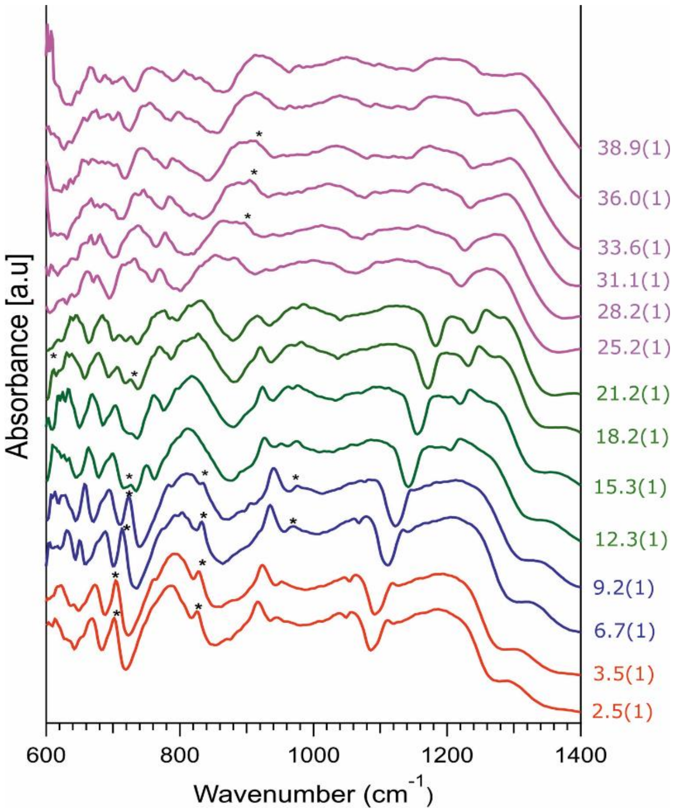


Fig. S6. Evolution of infrared spectra of danburite along the compression. Stars indicate peaks possibly originating from interferences of the beam between the diamond surfaces. The pressure is given in GPa. Different colours correspond to the high-pressure phases with distinct vibration properties.

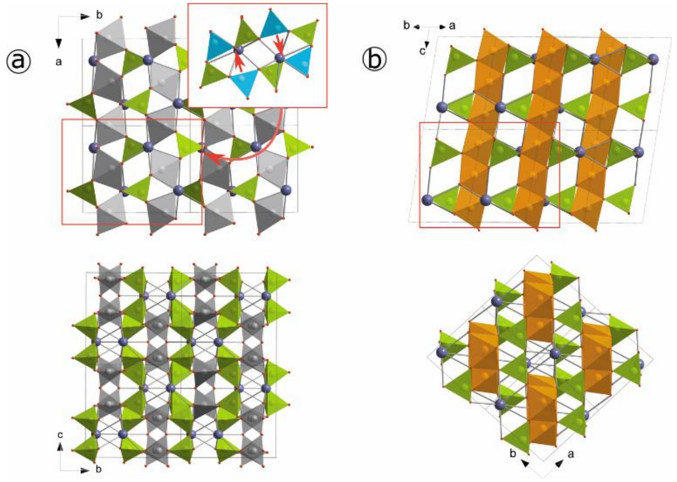


Fig. S7. The crystal structures of high-pressure phases of danburite. (a) The danburite-II (*Pnam*) with five-fold coordinated silicon in trigonal-bipyramidal geometry. The insert shows the mechanism of danburite → danburite-II phase transformation, *i.e*. a closure of eight-membered rings at pressures above 22.6(1) GPa. (b) The danburite-III (*P*-1) with octahedrally coordinated silicon. Grey, orange and green polyhedra represent $SiO_5$, $SiO_6$ and $BO_4$, respectively.

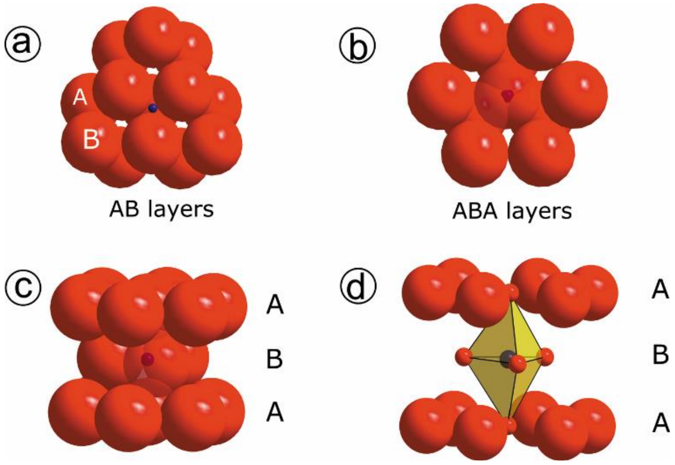


Fig. S8. Pentacoordinated void in a hexagonal close packing with sequence of layers ...ABAB... (a) View of two oxygen layers AB perpendicular to the stacking direction. The blue sphere is located between three oxygens in a layer B and above one oxygen from layer A. (b) A third oxygen layer A is located above layers AB so that now the blue sphere is positioned between five oxygens: three oxygens of layer B and two oxygens from two layers A from below and above. (c) View of the layers ABA along the stacking direction. (d) The coordination polyhedron of the blue sphere possesses geometry of trigonal bipyramid. For simplicity, size of spheres located in the vertices of trigonal bipyramid is reduced.

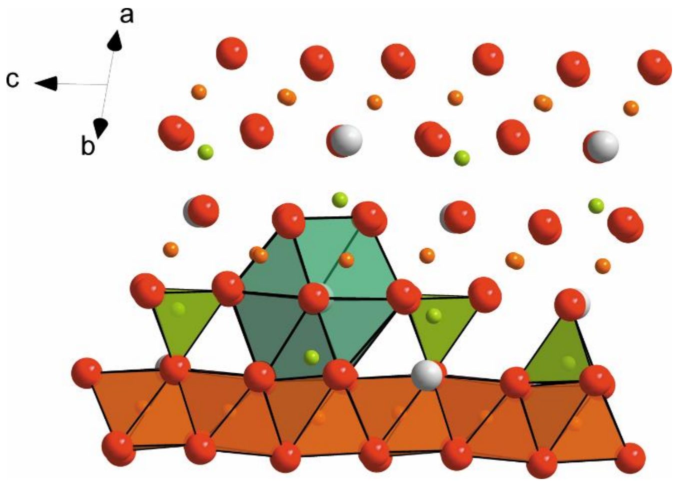


Fig. S9. O and Ca atoms (red and white, respectively) build cubic close packing in the structure of danburite-III (*P*-1). Si atoms (given in orange) occupy octahedral voids while the B atoms (given in green) are placed in tetrahedral voids. $CaO_{12}$ cubooctahedron is shown in blue.

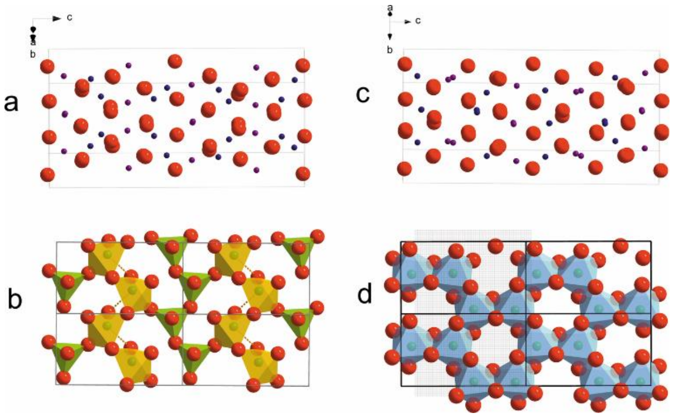


Fig. S10. The evolution of close packing of oxygen atoms in the structure of postorthoenstatite (Finkelstein et al., 2015) and corresponding change in silicon coordination. (a) The … ABCBACBC… packing of oxygen layers in alpha-postorthoenstatite. (b) The formation of distorted octahedral voids by displacement of oxygen layers. Note the displacement of Si atoms from the centre of the quasi-octahedral voids; the corresponding long non-bond contact is shown as a dashed line. (c) The hexagonal ..AB.. close packing in the structure of beta-postorthoenstatite . (d) The silicon atoms occupy octahedral voids.
